# Development of multilayered polymer-BaSO_4_ composites for flexible and efficient lead-free X-ray shielding

**DOI:** 10.1038/s41598-026-37398-x

**Published:** 2026-02-14

**Authors:** Hager M. Y. Okda, Emad R. Sheha, Fouad Zahran, Mohamed A. Yousef, Tharwat I. Shaheen, Ahmed G. Hassabo

**Affiliations:** 1 Textile Research and Technology Institute, Pretreatment, and Finishing of Cellulose-based Fibres Department, National Research Centre (Scopus affiliation ID 60014618), 33 El-Behouth St. (former El-Tahrir str.), Giza, P.O. 12622 Dokki Egypt; 2 Experimental Nuclear Physics Department, Nuclear Research Center, Egyptian Atomic Energy Authority, Cairo, 13759 Egypt; 3 Cyclotron Facility, Egyptian Atomic Energy Authority, Cairo, 13759 Egypt; 4 Chemistry Department, Faculty of Science, Helwan University, Cairo, 11795 Egypt

**Keywords:** Gelatin-BaSO₄ dual composite, Lead-free radiation shielding, Multilayered fabrics, X-ray, Thermal stability, Attenuation efficiency, Chemistry, Engineering, Materials science

## Abstract

The widespread use of toxic lead in radiation shielding materials poses serious environmental and health concerns, necessitating the development of safer alternatives. This study addresses this challenge by developing innovative multilayered polymer composites as eco-friendly, lead-free materials for X-ray attenuation. Gelatin-BaSO₄ dual composite coatings were applied to polyester and cotton fabrics using the pad-dry method to create lightweight and flexible shielding materials. In methodology, the effects of BaSO₄ concentration (40–60%), coating layers (1–5), and low-energy X-rays (up to 60 keV) on attenuation efficiency were systematically examined. The results revealed that FTIR analysis confirmed physical interactions between gelatin and BaSO₄, while SEM micrographs showed uniform dispersion of BaSO₄ within the polymer matrix. Additionally, increasing BaSO₄ content and layer number markedly enhanced X-ray shielding performance, with a five-layer coating containing 60% w/w BaSO₄ achieving 84.73% attenuation at 60 keV. TGA results indicated that higher BaSO₄ loadings improved thermal stability, elevating decomposition temperatures to 498 °C. The coatings also exhibited hydrophobicity and retained adequate flexibility despite a slight reduction in tensile strength. In conclusion, these findings elucidate that BaSO₄-based multilayer composites offer a sustainable, efficient, and lead-free solution for radiation protection in medical, industrial, and nuclear applications.

## Introduction

The increasing use of ionizing radiation in medical, industrial, and nuclear applications has heightened the demand for effective and lightweight shielding materials. Traditional lead (Pb)-based shields, while effective, pose significant environmental and health risks due to toxicity, weight, and rigidity^1^. Consequently, there is a pressing need for sustainable, flexible, and high-performance alternatives that can provide comparable protection without the drawbacks of lead. workers in a variety of medical fields and industries require the use of contemporary, specialized fabrics to ensure their safety^2^. It’s interesting to note that textile fabric is essential for shielding against ionizing radiation. The most popular fabric types for nuclear protection clothing are twill or sateen woven cotton, polyester/cotton, or nylon/polyester textiles^3^.

The purpose of radiation shielding is to lower the radiation intensity at the object’s location by placing a body of material between the thing to be protected and a radiation source. Several materials can be used to make it^4^. The practical application of X-ray shielding clothes is dependent on the integration of high-Z elements with structural supporting matrix, such as silicon rubber, polymers, and textiles, because of their economic viability and substantial chemical stability^5^. Elements with strong attenuation capabilities against ionizing radiation include tungsten, bismuth, silver, gold, molybdenum, zirconium, lead, polonium, gadolinium, rhodium, and depleted uranium^6^. To date, several types of X-ray shielding composites have been developed. The first type includes the in-situ deposition of X-ray shielding particles into polymers.

Early studies of ionizing radiation shielding have been achieved^1,5–10^. Polyester composites with barite and tungsten (PBaW50) demonstrated superior shielding, achieving 99.88% protection at 81 keV, highlighting their potential for industrial, medical, and aerospace use^11^. A study developed X-ray shielding fabrics using PbWO₄ and Bi₂WO₆ nanoparticle coatings, which also showed strong antibacterial activity, especially with low PbWO₄ loadings^6^. T. Liu et al. developed a flexible MXene/PVA/PCM-BaSO₄ composite film via vacuum filtration, showing superior thermal management, EMI shielding, and radiation protection^1^. Concurrently, Kaew-on et al. developed lightweight, flexible poly(vinylidene fluoride-co-hexafluoropropylene (P(VDF-HFP)/BaSO₄ nanocomposites with enhanced hydrophobicity and moisture resistance for durable medical and industrial use^7^.

Barium Sulfate (BaSO₄) is a non-toxic, high-density material that has been widely recognized for its X-ray attenuation properties. Due to its high atomic number and density, BaSO₄ is capable of effectively attenuating X-rays, making it a suitable alternative to lead. Moreover, BaSO₄ is chemically stable, it is resistant to alkalis and acids and has excellent weathering resilience, inexpensive, and widely available, further enhancing its attractiveness as a component in radiation shielding materials. Additionally, high loading levels (weight ratio of 60%) of barium sulfate can be added to a polymer without noticeably altering the polymer’s physical characteristics^7,12,13^. However, the challenge lies in integrating BaSO₄ into a flexible and durable matrix that can be easily processed into various forms suitable for practical applications. In this regard, Du Haiyang developed multilayer composite films combining high-Z materials, achieving 96.3% X-ray shielding at 100 keV-comparable to a 0.5 mm lead apron and surpassing single-element films^5^.

Gelatin, a natural biopolymer derived from collagen, offers several advantages as a matrix material for composite development. It is biocompatible, biodegradable, and possesses excellent film-forming capabilities, which make it ideal for coating applications^14–16^. Additionally, gelatin is capable of uniformly dispersing BaSO₄ particles within its matrix, ensuring consistent X-ray attenuation across the material. The combination of BaSO₄ with a gelatin matrix has the potential to create a composite material that is not only effective at blocking X-rays but also environmentally friendly and safe for human use.

Hence, this study develops lightweight, flexible, lead-free multilayer polymer composites with high-Z fillers like BaSO₄, optimizing composition, layer structure, and filler distribution to achieve effective X-ray shielding while retaining mechanical performance, offering safer alternatives for healthcare, industry, and nuclear applications.

Although BaSO₄-filled polymer composites and multilayer lead-free shielding structures have been reported in earlier studies, most prior work has focused on films, membranes, or rigid composite sheets that are not directly integrated into textile substrates. To date, there has been limited investigation into scalable textile finishing techniques capable of depositing BaSO₄-based shielding media onto flexible fabrics suitable for wearable protection. Furthermore, existing studies generally examine a single fabric type, without addressing how differences in fabric architecture affect coating uptake, attenuation performance, or mechanical behavior. The present study addresses these gaps by applying a pad-dry coating process—an industry-compatible and textile-friendly finishing method—to three distinct knitted fabric substrates (cotton, polyester, and cotton/polyester). This enables a systematic comparison of substrate-dependent shielding and flexibility characteristics, offering a practical pathway toward lightweight, flexible, and manufacturable lead-free protective textiles.

## Materials and methods

### Materials

BaSO_4_ powder was purchased from Fischer with 4.5 g/cm^3^ density 26 were employed for the study, Gelatin powder was purchased from Sigma Aldrich (CAS No.: 9000-70-8, code: G9391-500G). Polyester and cotton fabrics with different construction as illustrated in Table 1 were purchased from El-Mahala Company for spinning and weaving, Egypt.

**Table 1:** Base fabrics properties.

Number	1	2	3
Sample	Polyester	Polyester/Cotton	Cotton
Blended ratio	100%	(65:35) %	100%
Technique	Circular knitting	Circular knitting	Circular knitting
Structure	Single jersey	Single jersey	Single jersey
Warp/wales per cm	28 gauge for machine cylinder	28 gauge for machine cylinder	28 gauge for machine cylinder
Weft/courses per cm			
Warp yarn count	70/1	60/1	60/1
Weft yarn count			
Weight (g/m^2^)	140	170	190

### Preparation of Gelatin-BaSO₄ composite coating

**Fig. 1:**
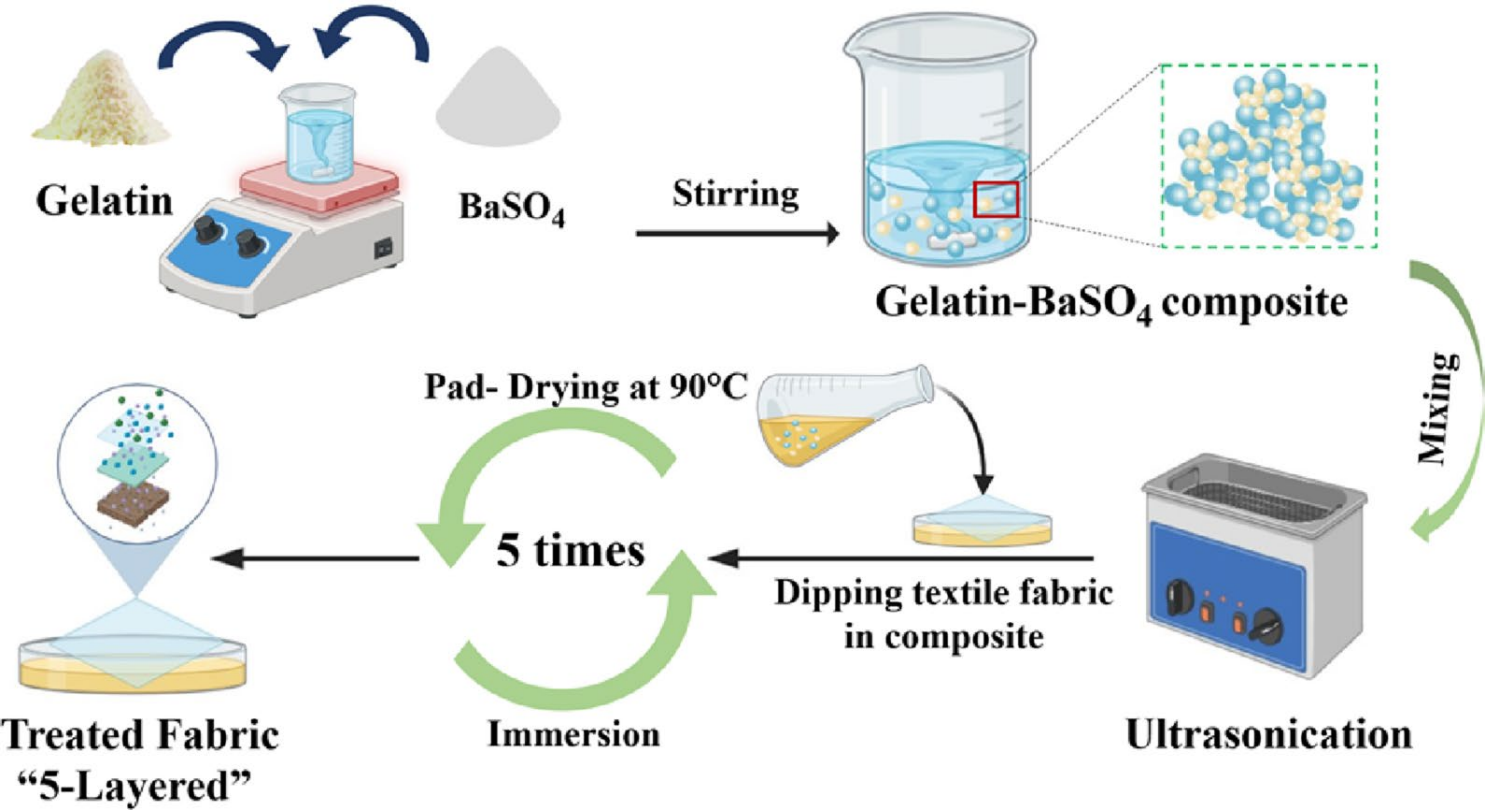
Ultrasound-assisted gelatin-BaSO₄ dual composite coating preparation layout.

Gelatin-BaSO₄ composite coating was developed via one pot synthesis method^6^, with some adjustments. In a word, gelatin was prepared by dissolving it in distilled water at a controlled temperature (45 °C) using a controlled water bath to form a homogeneous solution using different concentrations (2, 5, 7, 9% w/v). Meanwhile, BaSO₄ powder (6, 8, 10, 12% w/v) was gradually added to the gelatin solution while stirring continuously to ensure uniform dispersion. After that, ultrasonication water bath for 15 min was employed to further disperse the BaSO₄ particles and prevent agglomeration. Figure 1 illustrates the preparation of gelatin-BaSO₄ dual composite coating system assisted by ultrasound.

### Coating process

Gelatin-BaSO₄ dual complex mixtures were coated onto the fabric substrates using a pad dry coating technique. The suspended mixture was stirring for 30 min, under constant stirring in order to obtain a uniform distribution of BaSO4 inside the gelatin network. Then, the fabrics (10 × 10 cm^2^) were immersed in a mixture for 15 min at 60 °C, padded with pick up 100% and dried at 120 °C for 5 min^17^. Concurrently, the multilayer assembly process involved stacking many layers of coated fabric in different configurations to maximize both X-ray shielding effectiveness and mechanical qualities. The coating phase was done three times for each concentration in order to create a fabric treated with layers applied one after the other (mono-layer, bi-layer, till five layer).

### X-ray characterization

The experiment was conducted using an X-ray generator (Toshiba E7239, 150 kV–500 mA, 1999, Tokyo, Japan). The operational parameters were set at a current of 10 mA, an accelerating voltage of 100 keV, and an exposure time of 60 s, resulting in an absorbed dose of 1 Gy (J/kg). The applied irradiation energy ranged from low-energy X-rays (up to 60 keV), depending on the selected tube voltage (0, 20, 40, and 60 keV).

### FTIR measurements

The samples were mixed with KBr at a ratio of 1:100 (w/w), then ground and pressed into transparent tablets for infrared scanning according to the methodology of^18^. The scanning range was 400–4000 cm^− 1^, with a resolution of 4 cm^− 1^ and 32 accumulated scans. The air scan was subtracted as background before scanning the sample. FTIR spectra were obtained using a Nexus-470 FTIR spectrometer (Thermo Nicolet Corporation, Madison, USA).

### Mechanical properties measurement

The mechanical properties of the samples were evaluated according to the National Standard (GB/T 22890 − 2008, China). Tensile strength tests were performed using a universal testing machine (AI-7000 S, Changchun, China). The thickness (d, cm) of the samples was measured with a digital fabric thickness meter (YG141D, Shanghai, China) following the GB/T 24218.2–2009 standard.

### Scanning electron microscope (SEM) measurements

The microstructure of the treatments were performed using a scanning electron microscope (SEM, EVO 10, Tokyo, Japan) with an acceleration voltage of 20 kV after sputtering the samples with thin gold layer^19^.

### Thermogravimetric analysis (TGA)

The thermal stability of the samples was examined using a Q500 thermogravimetric analyzer (TA Instruments, New Castle, DE, USA) based on the method of^20^. Measurements were carried out under a continuous flow of high-purity nitrogen (99.99%) at 60 mL/min to maintain an inert environment and suppress oxidative reactions. Approximately 10 mg of each sample was placed in standard 100 µL aluminum pans, which were hermetically sealed and positioned in the autosampler. The samples were heated from 50 °C to 650 °C at a constant rate of 10 °C/min. Data collection and interpretation were conducted with Universal Analysis software (version 4.5 A, TA Instruments).

### Contact angle measurements

The contact angle of the samples was measured using the sessile drop technique with an optical goniometer (DSA25 100 W, Kruss, Hamburg, Germany), following the protocol of^21^. A layer film with a diameter of approximately 10 mm was evenly spread on a glass slide. A 5 µL droplet of distilled water was then carefully placed onto the film surface using a syringe. The droplet image was captured by a camera, and its shape was analyzed to calculate the contact angle based on the Laplace–Young equation.

### Statistical analysis

The results of the experiments were presented as the mean ± standard deviation (SD) of three replicates. Data were analyzed using SPSS Statistics 19.0 (IBM Corporations, New York, USA), and *p* < 0.05 was considered statistically significant. The graphs were made using Origin 2025 software (Origin Lab Inc., Northampton, MA, USA).

## Results and discussion

### FTIR analysis

**Fig. 2:**
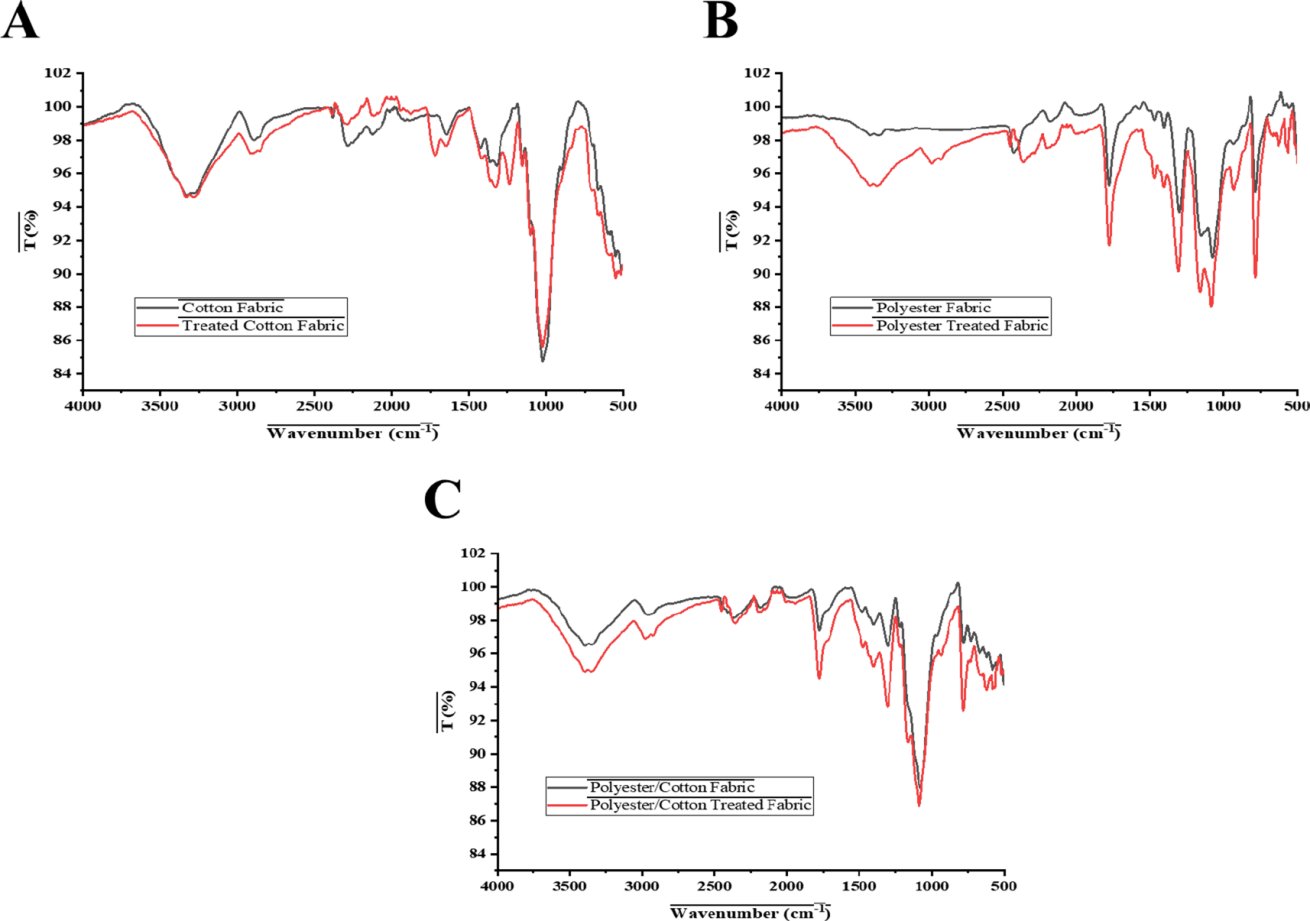
FTIR spectra for treated and untreated cotton, polyester, and cotton/polyester fabrics with gelatin-BaSO_4_ dual composites.

FTIR spectroscopy was used to reveal the chemical structure and potential interactions between the fabric substrates (cotton, polyester, cotton/polyester) and the applied gelatin-BaSO₄ treatment. As shown in Fig. 2, the treated samples (red spectra) show clear changes in position and/or intensity compared to the untreated fabrics (black spectra) in all cases. These modifications show that gelatin and BaSO₄ were successfully deposited on or interacted with the textile surfaces. FTIR spectra of untreated cotton displayed the characteristic absorption bands of cellulose (Fig. 2A). The O–H stretching vibration of hydroxyl groups in cellulose is indicated by a prominent band at 3300 cm⁻¹. The C–H stretching band around 2900 cm⁻¹ further substantiates the polysaccharide backbone. The notable absorption between 1000 and 1150 cm⁻¹ in the fingerprint region is attributed to C–O–C stretching vibrations in the glucosidic bonds of cellulose^22^. Furthermore, the addition of BaSO₄ to gelatin reveals enhanced absorption capabilities, confirming the presence of the coating. The peaks at 1650 cm⁻¹ (amide I), 1550 cm⁻¹ (amide II), and 1240 cm⁻¹ (amide III) indicate the effective deposition of the proteinaceous matrix onto the cotton fibers. The sulfate vibrations indicate the presence of BaSO₄: a prominent band at approximately 1100 cm⁻¹ (symmetric stretching of SO₄²⁻) and another band around 600 cm⁻¹ (bending of SO₄²⁻). The untreated cotton lacks these peaks, indicating the presence of BaSO₄ on the fabric’s surface.

Meanwhile, FTIR spectra of untreated polyester fabric exhibits the characteristic absorption bands of polyethylene terephthalate (Fig. 2B). The pronounced C–H stretching band at around 2960 cm⁻¹ indicates the presence of aliphatic groups inside the polymer backbone. The ester carbonyl group exhibits a pronounced C = O stretching vibration at approximately 1715 cm⁻¹. Absorptions within the 1240–1100 cm⁻¹ range result from C–O stretching vibrations in ester bonds. These characteristics demonstrate that polyester possessed a distinct chemical signature. Additional peaks emerged following treatment with BaSO₄ included in gelatin, indicating the presence of the coating system. The bands at 1650 cm⁻¹ (amide I), 1550 cm⁻¹ (amide II), and 1240 cm⁻¹ (amide III, which coincides with polyester C–O) originate from gelatin. This indicated that the protein matrix was effectively applied to the polyester surface. These results were in accordance with those of^23,24^. The presence of BaSO₄ is evident from the sulfate absorptions: a robust band at 1100 cm⁻¹ (SO₄²⁻ symmetric stretching) and a significant peak at 600 cm⁻¹ (SO₄²⁻ bending). The intersection of gelatin amide bands with BaSO₄ sulfate signals in the polyester spectrum indicates the formation of a composite surface layer. The constancy of ester peaks (C = O and C–O) is significant, as it indicated that the bulk polymer structure remains intact, although the surface chemistry has undergone substantial alteration. The spectrum changes observed strongly indicate that BaSO₄ was effectively incorporated into a gelatin matrix on polyester fabric, aligning with the results of^25^.

Moreover, the untreated polyester/cotton blend exhibited spectral characteristics of both cellulose and polyester as depicted in Fig. 2C. The O–H stretching of hydroxyl groups in cellulose manifests as a wide band about 3300 cm⁻¹. The C–H stretching at approximately 2900 cm⁻¹ is same for both components. The prominent C = O absorption at approximately 1715 cm⁻¹ indicates the presence of the ester carbonyl group in polyester. The prominent peaks between 1000 and 1150 cm⁻¹ indicate C–O–C stretching in cellulose and ester linkages^26^. The application of BaSO₄ embedded in gelatin results in the emergence of additional peaks, indicating the successful deposition of the composite layer. The gelatin matrix is indicated by bands at 1650 cm⁻¹ (amide I), 1550 cm⁻¹ (amide II), and 1240 cm⁻¹ (amide III). Certain bands coincide with polyester C–O vibrations. Prominent sulfate absorptions indicate the incorporation of BaSO₄: a robust SO₄²⁻ symmetric stretch about 1100 cm⁻¹ and a SO₄²⁻ bending vibration at approximately 600 cm⁻¹^25^. Additionally, the detection of cellulose (O–H, C–O–C), polyester (C = O, C–O), gelatin (amide bands), and BaSO₄ (sulfate vibrations) indicates that the modified fabric surface comprises multiple material types. The O–H band near 3300 cm⁻¹ exhibits a slight broadening and diminished intensity, indicating that cotton hydroxyls and gelatin are engaging in hydrogen bonding, hence enhancing the stability of the coating. This finding was consistent with the results for cellulose and gelatin-based hydrogel composites^27^. In a nutshell, FTIR analysis provided compelling evidence that BaSO₄ particles were effectively incorporated into a gelatin layer on the polyester/cotton blend, resulting in a hybrid organic-inorganic surface structure.

### Shielding of X-ray treated fabrics evaluation

#### Thickness

As displayed in Table 2, the fabric thickness increased consistently with increasing both the number of layers and the concentration of BaSO₄. The thickness of polyester increased from around 0.47 mm (1 layer) to roughly 0.86 mm (5 layers). Polyester/cotton increased from around 0.40 mm to approximately 0.84 mm, while Cotton rose from approximately 0.54 mm to approximately 0.90 mm. The use of BaSO₄ significantly improved the thickness, particularly at elevated concentrations (40–60%). After five layers, Cotton with 60% BaSO₄ attained a thickness of 1.53 mm. The results indicated that BaSO₄ particles contributed to the coating layer and altered the density of the cloth’s packing. Structural characteristics differentiate fabrics from each other. For instance, cotton fabric exhibited the greatest thickness, indicating enhanced porosity and superior absorption of the coating. This behavior aligned with previous research demonstrating that Increasing of BaSO₄ loading the thickness significantly increased^7,28^. So, the incorporation of fillers led to dimensional changes in textile composites^29,30^.

While previous studies have demonstrated BaSO₄-containing films and nanocomposites for X-ray attenuation (e.g., Liu et al., Kaew-on et al., Du et al.), these works focus on film fabrication or polymer matrices rather than textile processing. Here, we implement an industrially-relevant pad-dry coating on knitted substrates and provide the first direct comparison of polyester, polyester/cotton and cotton knitted fabrics under identical multilayer coating and X-ray testing conditions to identify substrate-dependent uptake, attenuation, and mechanical trade-offs relevant to wearable shielding.

**Table 2:** Effect of BaSO_4_ concentrations and number of layers on fabric thickness.

Samples	BaSO_4_ W%	Thickness (mm)				
		1 Layer	2 Layers	3 Layers	4 Layers	5 Layers
Polyester fabric	0	0.47 ± 0.007	0.50 ± 0.013	0.66 ± 0.011	0.73 ± 0.001	0.86 ± 0.005
	20	0.45 ± 0.01	0.51 ± 0.01	0.66 ± 0.005	0.79 ± 0.002	0.92 ± 0.003
	40	0.50 ± 0.01	0.53 ± 0.011	0.69 ± 0.010	0.77 ± 0.002	0.91 ± 0.005
	60	0.52 ± 0.009	0.55 ± 0.013	0.84 ± 0.013	0.94 ± 0.015	0.98 ± 0.005
Polyester/Cotton fabric	0	0.4 ± 0.006	0.43 ± 0.009	0.66 ± 0.004	0.71 ± 0.005	0.84 ± 0.002
	20	0.42 ± 0.008	0.44 ± 0.012	0.66 ± 0.006	0.73 ± 0.005	0.89 ± 0.008
	40	0.44 ± 0.007	0.46 ± 0.009	0.67 ± 0.005	0.75 ± 0.003	0.89 ± 0.007
	60	0.46 ± 0.007	0.48 ± 0.01	0.73 ± 0.006	0.81 ± 0.006	0.90 ± 0.008
Cotton fabric	0	0.54 ± 0.008	0.58 ± 0.012	0.7 ± 0.006	0.74 ± 0.006	0.90 ± 0.008
	20	0.56 ± 0.009	0.59 ± 0.012	0.83 ± 0.007	0.88 ± 0.007	0.95 ± 0.009
	40	0.58 ± 0.009	0.61 ± 0.012	0.89 ± 0.007	0.95 ± 0.007	1.05 ± 0.010
	60	0.61 ± 0.009	0.64 ± 0.013	0.98 ± 0.008	1.08 ± 0.008	1.53 ± 0.014

All data represent the mean of triplicates ± standard deviation (SD).

The correlation between BaSO₄ concentration, the number of coating layers, and the resultant thickness did not exhibit a perfectly proportional trend (Table 2). This nonlinearity aligns with established behavior in particulate-filled textile coating systems and results from multiple interacting factors. Initially, knitted fabrics exhibit variations in pore structure, yarn crimp, and surface energy, resulting in inconsistent absorption of the gelatin–BaSO₄ suspension across successive coating cycles. Following the initial and secondary layers, incomplete pore filling and surface saturation inhibit additional penetration, leading to progressively smaller increments in thickness. Secondly, elevated BaSO₄ concentrations enhance slurry viscosity, facilitating surface deposition while concurrently diminishing capillary-driven wicking into the fabric’s interior, resulting in uneven accumulation across layers. Third, ultrasonic dispersion generates microscale heterogeneity in particle distribution, and during the drying process, localized aggregation or movement of BaSO₄ inside the gelatin matrix may result in minor fluctuations in layer compaction. Comparable nonlinear thickness responses have been documented in polymer–ceramic and textile finishing systems, where substrate architecture and filler–matrix interactions prevail over the nominal add-on quantity. Consequently, the observed non-proportionality does not signify data inconsistency but rather illustrates the genuine, substrate-dependent deposition kinetics of multilayer particle coatings. Further rheological and penetration-depth imaging investigations are scheduled to enhance the quantification of these mechanisms.

#### Radiation Attenuation efficiency (AE)

Table 3 illustrates the impact of BaSO₄ concentration and the number of layers on AE. For polyester, the absorption efficiency increased from approximately 30% (0%, 1 layer) to roughly 73% (60%, 5 layers). Polyester/cotton improved from approximately 26% (0%, 1 layer) to around 65% (60%, 5 layers). Cotton exhibited the highest efficiency, increasing from approximately 35% (0%, 1 layer) to almost 85% (60%, 5 layers). The results indicated that BaSO₄ was a significant high-Z filler that enhanced photon scattering and absorption. The multilayer stacking exhibits a synergistic effect, particularly at 60% BaSO₄, where shielding exceeded 80% for fabric 3. The superior performance of cotton further underscores the significance of the fabric substrate. Cotton retained the coating and encapsulated the filler more effectively than synthetic and hybrid fabrics. Prior research has also demonstrated that the use of BaSO₄ and multilayered fabrics can enhance shielding in analogous manners^7,31^.

**Table 3:** Effect of BaSO_4_ concentrations and number of layers on Attenuation efficiency (AE) at 60 keV X-ray dose.

Fabrics	BaSO4 W%	AE (%)				
		1 Layer	2 Layers	3 Layers	4 Layers	5 Layers
Polyester fabric	0	30.75 ± 0.474	31.51 ± 0.633	37.78 ± 0.301	40.43 ± 0.309	42.27 ± 0.39
	20	47.15 ± 0.726	52.42 ± 1.053	56.29 ± 0.449	57.65 ± 0.441	58.65 ± 0.541
	40	61.88 ± 0.953	61.25 ± 1.231	65.87 ± 0.526	63.78 ± 0.488	67.88 ± 0.627
	60	67.11 ± 1.034	67.24 ± 1.351	69.89 ± 0.558	71.23 ± 0.545	73.24 ± 0.676
Polyester/Cotton fabric	0	26.36 ± 0.406	27.33 ± 0.549	36.82 ± 0.294	39.4 ± 0.301	41.18 ± 0.38
	20	40.87 ± 0.629	44.92 ± 0.903	48.25 ± 0.385	50.76 ± 0.388	57.32 ± 0.529
	40	53.04 ± 0.817	52.5 ± 1.055	56.46 ± 0.451	52.44 ± 0.401	61.63 ± 0.569
	60	57.52 ± 0.886	57.64 ± 1.158	59.91 ± 0.478	61.06 ± 0.467	65.31 ± 0.603
Cotton fabric	0	35.14 ± 0.541	36.44 ± 0.732	38.75 ± 0.309	41.47 ± 0.317	43.35 ± 0.400
	20	54.49 ± 0.839	59.89 ± 1.204	64.34 ± 0.513	64.54 ± 0.494	68.75 ± 0.635
	40	70.73 ± 1.089	70.00 ± 1.407	75.29 ± 0.601	75.11 ± 0.574	78.44 ± 0.724
	60	76.69 ± 1.181	76.85 ± 1.545	79.88 ± 0.637	81.41 ± 0.623	84.85 ± 0.783

All data represent the mean of triplicates ± standard deviation (SD).

Conncurently, the impact of X-ray energy on AE was also investigated as shown in Table 4. Where AE decreased as X-ray energy increased. This aligned with the observation that the photoelectric effect was diminished, allowing higher-energy photons to penetrate more deeply^28,32^. At 60 keV, the shielding was most effective, with cotton (60% BaSO₄, 5 layers) obstructing around 84.8% of the light. As energy increased to 120 keV, efficiency decreased for all samples. Additionally, the polyester and polyester/cotton fabrics exhibited the lowest values.

Despite this occurrence, multilayered samples containing significant amounts of BaSO₄ exhibited considerable shielding efficacy, indicating their potential application in protection against medium-energy X-rays. Cotton-based composites consistently exhibited superior attenuation at all energy levels, underscoring the importance of substrate structure in improving filler interaction and shielding effectiveness^7^. These findings agreed with those of^33^.

**Table 4:** Effect of X-ray energy and number of layers on Attenuation efficiency (AE) at 60% BaSO_4_.

Fabrics	X-Ray Energy (KeV)	AE (%)				
		1 Layer	2 Layers	3 Layers	4 Layers	5 Layers
Polyester fabric	0	67.11 ± 1.034	67.24 ± 1.351	69.89 ± 0.558	71.23 ± 0.545	74.25 ± 0.685
	20	47.23 ± 0.727	47.75 ± 0.733	51.22 ± 0.712	58.72 ± 0.811	62.72 ± 0.926
	40	32.17 ± 0.495	40.54 ± 0.815	48.16 ± 0.384	53.28 ± 0.407	58.41 ± 0.539
	60	16.72 ± 0.258	22.39 ± 0.45	27.97 ± 0.223	32.52 ± 1.648	41.82 ± 2.192
Polyester/Cotton fabric	0	57.52 ± 0.886	57.64 ± 1.158	59.91 ± 0.478	61.06 ± 0.467	63.64 ± 0.587
	20	40.49 ± 0.624	41.49 ± 0.834	43.1 ± 1.929	48.08 ± 0.368	54.05 ± 0.499
	40	27.58 ± 0.425	34.75 ± 0.698	44.35 ± 0.354	45.67 ± 0.349	50.07 ± 0.462
	60	14.33 ± 0.221	19.19 ± 0.386	23.98 ± 0.191	29.25 ± 0.224	33.97 ± 0.314
Cotton fabric	0	76.69 ± 1.181	76.85 ± 1.545	79.88 ± 0.637	81.41 ± 0.623	84.85 ± 0.783
	20	53.98 ± 0.831	54.67 ± 1.099	59.03 ± 0.471	62.59 ± 5.172	72.07 ± 0.665
	40	36.77 ± 0.566	46.33 ± 0.931	55.04 ± 0.439	60.89 ± 0.466	66.76 ± 0.616
	60	19.11 ± 0.294	25.59 ± 0.514	31.97 ± 0.255	39 ± 0.298	45.29 ± 0.418

All data represent the mean of triplicates ± standard deviation (SD).

As expected, the attenuation ratios of the structures decreased with increasing X-ray energy levels (Table 4). Moreover, the BaSO_4_ sample exhibited enhanced shielding capabilities with elevated concentrations of barium sulfate powder embedded in the sample, as illustrated in Table 3 for all three fabric types produced at varying powder weight ratios (20, 40, 60%, w/w). These results indicated that at the tested energy level of 60 KeV, the single layer of coated fabric offered inadequate protection.

#### Implication of layers number on mechanical properties

In this study, the constructs were arranged in a layered manner to enhance the overall thickness of the samples and improve shielding ratios. As the number of layers in the multilayered composites increased from 2 to 5, as illustrated in Fig. 5a, their X-ray attenuation efficiency rose significantly. The attenuation efficiency rose dramatically with the number of layers. In a similar manner, when high-atomic-number (high-Z-number) metallic particles were placed in front of the multilayered composites, resulting in the X-rays initially striking them, the extent of photon attenuation also increased. The multilayered structure augmented the likelihood of the photoelectric effect manifesting in different locations inside the composites. Additionally, it resulted in the X-rays being reflected and absorbed between the layers several times, which further dissipated the photon energy^34^. Moreover, Compton scattering causes the X-rays to be reflected when they interact with the shielding material’s atoms, whereas the photoelectric effect produces secondary X-rays, which have a lower energy than the original X-rays^5^.

**Fig. 3:**
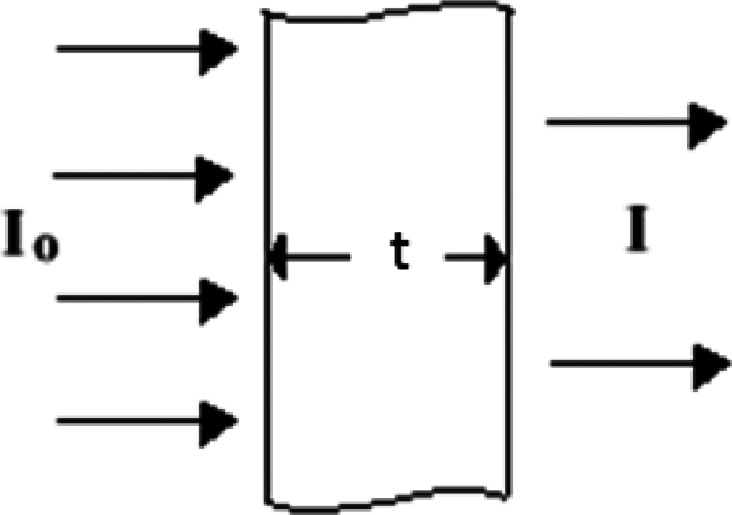
Illustration of radiation attenuation through material.

Figure 3 shows how radiation is blocked by a shielding material, following the exponential law.

$${\mathrm{I}} = {\mathrm{I}}_{{\mathrm{o}}} \times {\mathrm{e}}^{{ - \mu {\mathrm{t}}}}$$

Where I_o_ is the starting beam intensity and I is the intensity that passes through the fabric. µ represents the linear attenuation coefficient of the fabric (cm^− 1^) and t denotes the thickness of the fabric (cm).

This relationship showed that increasing the thickness or the attenuation coefficient makes the radiation that passes through it drop off very quickly. The attenuation efficiency (AE) of both untreated and BaSO₄/gelatin-treated fabrics was determined using the following formula:

$$\:AE=1-\frac{I}{Io}\times100$$

In this regard, adding BaSO₄ nanoparticles to the fabric makes it much more µ because barium has a high atomic number and density. This improves the fabric’s ability to protect. Furthermore, the use of many layers enhances attenuation by significantly elevating thickness. As a result, the treated fabrics should have AE values that are much higher than those of untreated fabrics. This shows that adding BaSO₄ and stacking fabrics together makes radiation shielding more effective. Where cotton with 5-layered of 60% w ratio of coating showed excellent shielding ability greater than 80% bringing thickness of 1.5 mm (shielding rate % 84.73), consisting with the results of^35^.

#### Statistical evaluation of the experimental results

The statistical analysis of the experimental data provides substantial insights into the effectiveness of the treated fabrics as displayed in Table 5. The ANOVA results for thickness measurements indicated a significant disparity among the various layers (F = 58.55, *p* < 0.0001). The thickness of the fabrics is significantly influenced by the BaSO₄ ratio and the number of layers. Moreover, the average thickness increased with the number of layers, indicating that the textile structure became more dense. This agreed with previous studies indicating that the incorporation of inorganic fillers such as BaSO₄ into textile matrix alters the dimensional stability of the fabric^36,37^. The significant variances confirm that structural variation has a measurable impact on thickness and must be considered in material design optimization.

**Table 5:** ANOVA statistical results.

Dataset	F-statistic	*p*-value	Significance
Thickness	58.55	< 0.0001	Significant
AE	2.32	0.078	Not Significant
X-Ray energy	4.38	0.0055	Significant

In conclusion, Table 5 presented an overview of the comprehensive ANOVA results. Structural alterations significantly influenced thickness and X-ray attenuation characteristics, despite AE remaining statistically unchanged. This demonstrated an effective equilibrium, as increasing the layers enhanced the defensive attributes without compromising the visual appeal.

### Scanning electron microscope (SEM) analysis

**Fig. 4:**
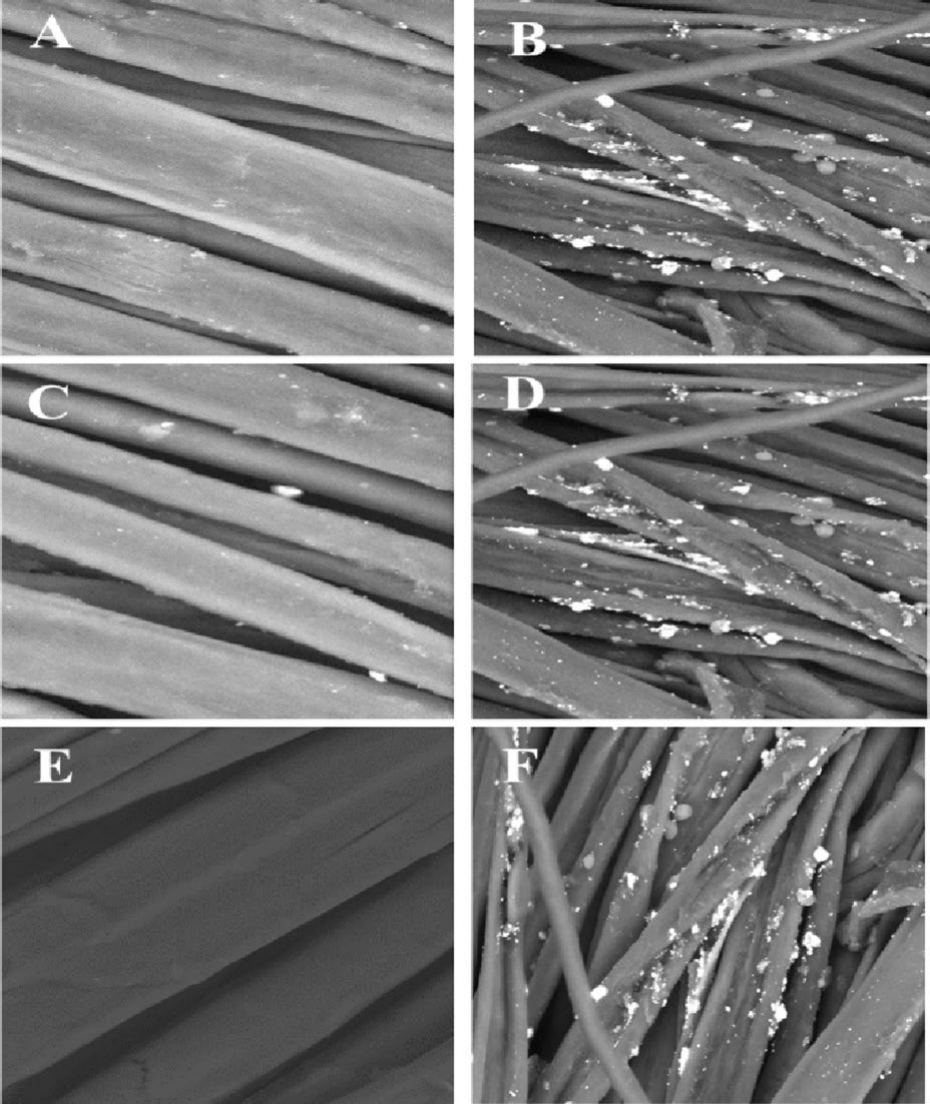
SEM micrographs for untreated and treated cotton, polyester, and cotton/polyester fabrics with gelatin-BaSO_4_ composites. (**A**) represents untreated cotton fabric; (**B**) denotes treated Cotton fabric. (**C**) is untreated polyester/cotton fabric; (**D**) is treated polyester/cotton fabric. (**E**) refers to untreated polyester fabric; and (**F**) represents treated polyester fabric.

SEM was employed to elucidate the microtopography of the samples. Figure 4 shows the surface of the untreated fabric, characterized by imperfections and minute perforations. The coated sample demonstrates that the coating effectively fills the gaps, pores, and interstices of fiber bundles while enveloping the fabric’s surface and forming a protective layer^10^. The coating penetrated the structure of the coated fabric, resulting in a durable finish. As also illustrated in Fig. 4, the distribution of barium sulfate (BaSO₄) particles within the polymer matrix of the treated cotton showed that the particles were at the micron scale and that the polymer-BaSO₄ coating was applied uniformly across the fabric surface.

The SEM micrographs of untreated cotton (Fig. 4A) revealed smooth, elongated fibers with natural grooves and ridges characteristic of cellulose, displaying only minor surface irregularities typical of pure cotton. In contrast, the treated cotton (Fig. 4B) exhibited a markedly different morphology. The fibers are coated with a coarse, granular layer, where BaSO₄ particles appear as evenly distributed spherical and irregular deposits embedded within the gelatin matrix. This coating conceals much of the cotton’s intrinsic surface structure, indicating effective surface functionalization^38^. Furthermore, the deposition of BaSO₄ increased the surface roughness and microstructural variability, which enhanced the fabric’s ability to block UV radiation, improve hydrophobicity, and acted as a functional barrier. Gelatin served as a binding matrix, ensuring strong adhesion between the inorganic filler and cellulose fibers^39^. Overall, the BaSO₄-gelatin treatment transformed the cotton fibers into a composite structure with enhanced functional properties compared to untreated fabric.

As illustrated in Fig. 4C, the untreated polyester/cotton fabric showed smooth and uniform fibers with minimal surface irregularities, reflecting the natural morphology of the blend. In contrast, the treated fabric (Fig. 4D) exhibited dense coverage of irregular particles embedded in a matrix, increasing surface roughness and forming a continuous coating in some areas. This modification confirmed successful deposition and enhanced functional properties such as barrier performance, UV protection, and flame retardancy^40^.

The SEM image of untreated polyester (Fig. 4E) showed smooth, homogeneous filaments typical of synthetic polyester, with a dense arrangement and minimal surface irregularities, reflecting the absence of coatings or fillers. In contrast, polyester treated with BaSO₄ embedded in gelatin (Fig. 4F) exhibited a marked transformation, where the filaments were coated with spherical and irregular BaSO₄ particles firmly embedded in the gelatin matrix, resulting in increased surface roughness and uneven particle distribution. This dense coverage obscures the fibers’ natural smoothness, confirming successful deposition of the inorganic-organic coating^41^. In addition, the morphology indicated strong adhesion of the BaSO₄-gelatin composite to the polyester surface, despite its inert and hydrophobic nature. The enhanced roughness and particle coverage are expected to improve fabric functionalities such as UV protection, flame retardancy, and barrier properties, while gelatin ensures effective compatibility between the inorganic filler and polymer substrate^23^. In summary, SEM analysis indicated that the BaSO₄-gelatin treatment significantly altered polyester fibers, resulting in a durable surface coating that was distinctly different from the untreated condition, hence presenting evident promise for multifunctional textile applications.

### Thermal stability analysis

**Fig. 5:**
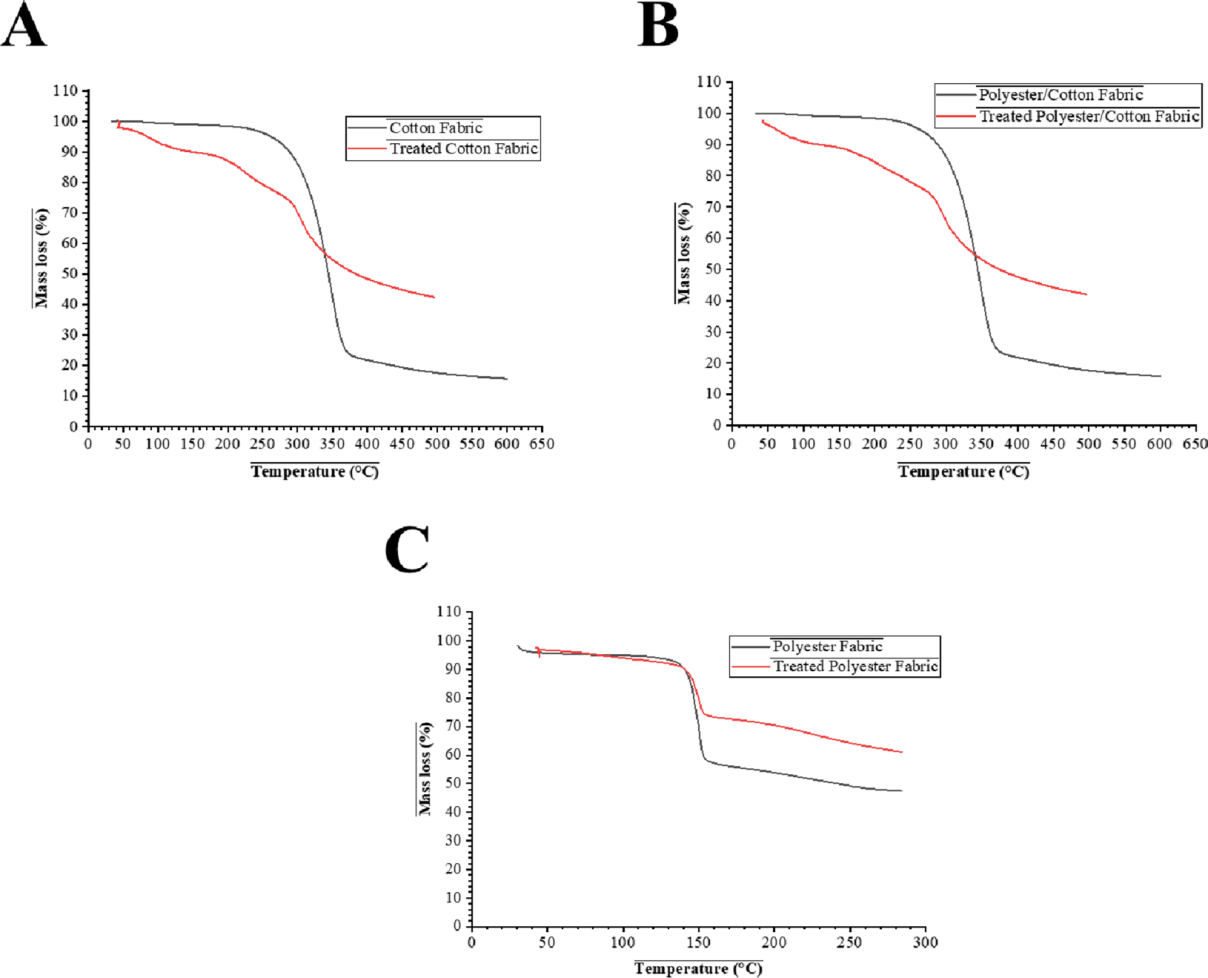
TGA thermograms of untreated and treated fabrics with gelatin-BaSO_4_ composites (**A**) cotton; (**B**) cotton/polyester; and (**C**) polyester fabrics.

The thermal stability of both untreated and treated fabrics was assessed using TGA, with the findings presented in Fig. 5; Table 6. Untreated cotton and cotton/polyester fabrics exhibited characteristic cellulose disintegration patterns, with a beginning around 280 °C, fast degradation occurring between 300 and 370 °C, and minimal final residues (~ 17–18%). Conversely, the treated equivalents demonstrated an earlier onset (~ 200 °C), due to the first disintegration of gelatin, yet exhibited a significantly higher char production (~ 40%). This rise could be attributed to the synergistic impact of gelatin, which facilitated char formation, and BaSO₄, which persisted as a thermally persistent inorganic residue^7,42^.

Additionally, the untreated polyester fabric exhibited a singular primary breakdown phase commencing about 380 °C, resulting in minimal residue above 300 °C. Post-treatment, the polyester demonstrated an earlier commencement of disintegration (~ 200 °C) alongside a markedly increased residue (~ 62% at 300 °C in contrast to ~ 48% for the untreated sample). This suggested that the gelatin-BaSO₄ dual system coating significantly improved the char-forming propensity of polyester, which usually decomposed nearly entirely into volatiles^43^. Concurrently, the 60% BaSO₄ composite showed the highest residual mass, increasing with higher BaSO₄ content. This stability stems from thermally resistant BaSO₄, which maintains structural integrity after polymer degradation, enhancing suitability for high-temperature use. BaSO₄ remains stable up to about 1580 °C, with decomposition behavior depending on the surrounding atmosphere^44^. As shown in Table 6, the treated fabrics exhibited enhanced thermal stability at high temperatures and markedly increased char yields, underscoring the flame-retardant efficacy of the gelatin-BaSO₄ treatment. The findings validate that the modification operates via the establishment of a physical barrier by BaSO₄ and the enhancement of char formation by gelatin, significantly diminishing the total combustion of cotton, polyester, and their composite^45,46^.

**Table 6:** TGA parameters for untreated and treated fabrics.

Sample	Onset Temp (°C)	Tmax (°C)	Residue (%)	Notes
Cotton (untreated)	~ 280 ± 5	~ 350 ± 5	~ 18	Rapid degradation, low char yield
Cotton (treated)	~ 200 ± 5	~ 330 ± 10	~ 40	Early gelatin loss, strong char yield (gelatin + BaSO₄)
Polyester/Cotton (untreated)	~ 280 ± 5	~ 350 ± 10	~ 17	Combined cellulose + polyester peaks
Polyester/Cotton (treated)	~ 200 ± 5	~ 330 ± 10	~ 40	Higher char due to BaSO₄, stabilized blend
Polyester (untreated)	~ 380 ± 5	~ 430 ± 10	~ 48 (at 300 °C)	Typical polyester decomposition
Polyester (treated)	~ 200 ± 5	~ 430 ± 10	~ 62 (at 300 °C)	Strong increase in char at 300 °C

### Contact angle analysis

For X-ray shielding materials to function well over the long term, surface wettability is essential, especially in moist settings like industrial applications, protective clothing, and medical devices. Over time, hydrophobic surfaces assist preserve the composite material’s mechanical integrity and shielding effectiveness by preventing water absorption^47^. Degradation of the polymer matrix, weakened mechanical strength, and decreased radiation attenuation efficiency can all result from moisture infiltration. One of important measures of the samples’ wettability is the contact angle. A surface’s ability to resist or attract water is indicated by itsWater contact angle (WCA), which is below 90◦ for hydrophilicity, between 90◦ and 150◦ for hydrophobicity, and over 150◦ for superhydrophobicity^47^.

**Fig. 6:**
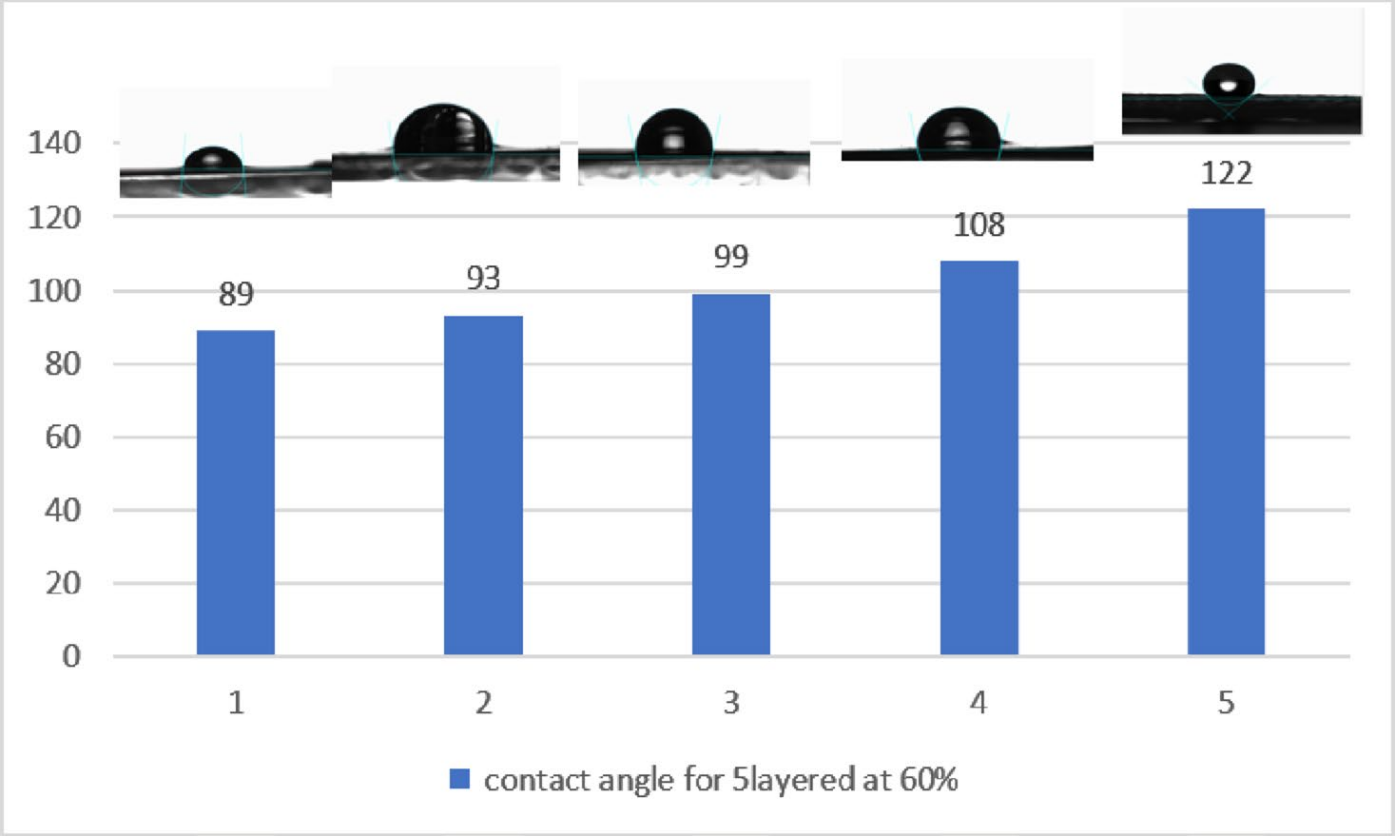
Contact angle for 5 layered treated fabrics.

As shown in Fig. 6, increasing the number of treatment layers raised the WCA from 89° to 122°, indicating a shift from hydrophilic to hydrophobic behavior. The five-layer BaSO₄-gelatin composite coating ultimately imparted super hydrophobicity to the fabric, with a contact angle exceeding 120°. In addition, the enhanced hydrophobicity results from increased surface roughness due to additional BaSO₄ coating layers and reduced surface energy from chemical modifications in BaSO₄ and gelatin^48^. With more uniform layer deposition, higher contact angles are achieved, indicating improved water repellency, which is beneficial for self-cleaning, moisture-resistant, and protective textile applications^49^.

### Fabric weight analysis

Fabric weight is strongly influenced by filler content, layering, and material composition. As depicted in Table 7, the fabric weight increased consistently with higher BaSO₄ concentrations, additional layers, and fabric type. Among the materials, cotton absorbed the most, followed by polyester/cotton blends, with polyester showing the least uptake due to its smooth, low-absorbency fibers. For polyester, untreated fabrics gained little weight with layering, but 60% BaSO₄ in five layers increased mass to 402 g/m². Blends showed intermediate behavior, reaching 536 g/m² at 60% BaSO₄, reflecting enhanced absorption from the cotton fraction but limited by polyester. Cotton exhibited the most dramatic increase, with five-layer, 60% BaSO₄ samples reaching 1060.5 g/m², nearly nine times the weight of untreated single-layer fabric. This was due to its hydrophilic, porous, and coarse structure, which promotes BaSO₄ deposition^50^. Additionally, layering amplified weight gains and variability, with cotton demonstrating the highest sensitivity to filler content. Overall, cotton is ideal for applications demanding high weight and bulk, such as radiation shielding, while polyester offers lighter, more flexible options. Polyester/cotton blends provide a balanced compromise between weight, filler retention, and flexibility. These findings were in agreement with those of^38^, who demonstrated that fabric weight was significantly influenced by filler content, layering, and material composition.

**Table 7:** Weight of samples of fabrics treated with different layers using various BaSO_4_ concentrations.

Fabrics	BaSO4 W%	Weight (g/m^2^)				
		1 Layer	2 Layers	3 Layers	4 Layers	5 Layers
Polyester	0	61.3 ± 0.944	62.89 ± 1.264	67.98 ± 0.542	70.16 ± 0.537	74.84 ± 0.691
	20	68.11 ± 1.049	108.29 ± 1.663	117.86 ± 1.639	123.68 ± 1.709	133.4 ± 1.971
	40	136.21 ± 2.098	215.86 ± 4.339	237.72 ± 1.897	248.84 ± 1.903	268.25 ± 2.476
	60	204.32 ± 3.147	323.79 ± 6.508	356.58 ± 2.845	373.26 ± 2.855	402.38 ± 3.715
Polyester/Cotton	0	74.24 ± 1.143	76.16 ± 1.531	82.34 ± 0.657	84.97 ± 0.65	90.64 ± 0.837
	20	82.49 ± 1.27	131.89 ± 2.651	97.3 ± 48.845	154.84 ± 1.184	178.63 ± 1.649
	40	164.97 ± 2.541	263.78 ± 5.302	263.9 ± 14.346	309.68 ± 2.368	357.27 ± 3.298
	60	247.46 ± 3.811	395.68 ± 7.953	377.1 ± 3.009	464.52 ± 3.553	535.9 ± 4.947
Cotton	0	103.63 ± 1.596	106.32 ± 2.137	114.93 ± 0.917	118.61 ± 0.907	126.52 ± 1.168
	20	115.14 ± 1.773	229.15 ± 4.606	223.93 ± 1.787	299.29 ± 2.257	353.5 ± 3.263
	40	230.29 ± 3.547	458.3 ± 9.211	447.86 ± 3.573	589.67 ± 4.51	707 ± 6.527
	60	345.43 ± 5.32	687.44 ± 13.87	671.79 ± 5.36	884.51 ± 6.765	1060.5 ± 9.79

All data represent the mean of triplicates ± standard deviation (SD**)**.

### Fabric stiffness analysis

Fabric stiffness is a critical property for applications requiring structural integrity and durability. As shown in Table 8, the addition of BaSO₄ combined with layering markedly increased fabric rigidity. While control samples (0% BaSO₄) showed minimal change, fabrics with BaSO₄ exhibited a sharp rise, especially above 40% loading. For instance, five layers of cotton with 60% BaSO₄ reached ~ 2500 units, compared to ~ 42 units for untreated cotton exhibited reduced flexibility and are better suited for semi-flexible panel applications. This demonstrated that BaSO₄ not only added weight but also acted as a rigid filler, restricting fabric flexing^51^. Moreover, cotton showed the highest stiffness due to better filler penetration and bonding, polyester exhibited minimal stiffening, and polyester/cotton blends were intermediate. These results were consistent with the reported that the incorporation of BaSO₄ into fabrics significantly enhanced their stiffness and rigidity, making them suitable for structural and protective applications. In a word, BaSO₄ transformed fabrics into rigid composites, with effectiveness depending on fabric type, serving as a strong reinforcing agent for cotton and a moderate stiffener for polyester, making it suitable for structural or protective applications, though potentially reducing comfort. Afterall, the fabrics remain bendable and drapable, particularly at BaSO₄ contents ≤ 40% and ≤ 3 layers, which correspond to the property window suitable for wearable shielding (e.g., lightweight aprons, vests, sleeves).

**Table 8:** Stiffness property of fabrics treated with different layers using various BaSO_4_ concentrations.

Fabrics	BaSO4 W%	Stiffness				
		1 Layer	2 Layers	3 Layers	4 Layers	5 Layers
Polyester fabric	0	5.95 ± 0.092	11.63 ± 0.234	17.96 ± 0.143	23.54 ± 0.18	29.89 ± 0.276
	20	119.05 ± 1.833	233.41 ± 3.584	356.26 ± 4.954	467.99 ± 6.467	594.65 ± 8.784
	40	238.09 ± 3.667	465.27 ± 9.351	718.54 ± 5.733	941.59 ± 7.201	1195.74 ± 11.038
	60	357.14 ± 5.5	697.9 ± 14.027	1077.82 ± 8.6	953.68 ± 796.189	1210.46 ± 1014.351
Polyester/Cotton fabric	0	8.93 ± 0.138	23.26 ± 0.468	29.94 ± 0.239	35.31 ± 0.27	41.85 ± 0.386
	20	178.57 ± 2.75	465.27 ± 9.351	412.16 ± 321.545	706.19 ± 5.401	837.02 ± 7.727
	40	357.14 ± 5.5	930.54 ± 18.703	1257.11 ± 68.34	1412.38 ± 10.802	1674.03 ± 15.454
	60	535.71 ± 8.251	1395.8 ± 28.054	1796.36 ± 14.333	2118.58 ± 16.203	2511.05 ± 23.181
Cotton fabric	0	10.42 ± 0.16	31.45 ± 0.632	35.57 ± 0.284	38.1 ± 0.291	41.9 ± 0.387
	20	208.33 ± 3.209	628.96 ± 12.641	711.48 ± 5.677	534.94 ± 414.263	838.02 ± 7.736
	40	416.66 ± 6.417	1257.92 ± 25.283	1422.96 ± 11.353	1524.13 ± 11.657	1676.05 ± 15.472
	60	624.99 ± 9.626	1886.89 ± 37.924	2134.44 ± 17.03	2286.2 ± 17.485	2514.07 ± 23.208

All data represent the mean of triplicates ± standard deviation (SD**)**.

### Crease recovery angle analysis

Crease recovery angle (CRA) is a key indicator of a fabric’s ability to resist wrinkling and maintain its appearance during use, making it a critical parameter for assessing textile performance and durability. As illustrated in Table 9, both BaSO₄ concentration and the number of fabric layers significantly improved the CRA. Untreated polyester and cotton exhibited CRA values below 100°, while fabrics treated with 40–60% BaSO₄, particularly in multilayered cotton and polyester/cotton blends, reached angles of 200–400°. This demonstrated that BaSO₄ enhanced fabric stability by increasing fiber rigidity and limiting wrinkle formation. Furthermore, the effect was most pronounced in cotton, likely due to better filler penetration, while polyester/cotton blends benefited from a balance of filler absorption and structural reinforcement^38^. Layering further amplified the effect, strengthening the filler-fiber interface. Polyester, already resistant to wrinkling, showed only slight improvement, whereas cotton achieved substantial gains, with multilayered cotton at 60% BaSO₄ nearly matching the performance of engineered wrinkle-resistant textiles. In brief, BaSO₄ effectively counteracted cotton’s natural tendency to wrinkle by stiffening fibers and reducing cellulose chain mobility, with polyester/cotton blends showing intermediate enhancement.

**Table 9:** Crease recovery angle (CRA) of fabrics treated with different layers using various BaSO_4_ concentrations.

Fabrics	BaSO4 W%	CRA (W + W)				
		1 Layer	2 Layers	3 Layers	4 Layers	5 Layers
Polyester fabric	0	45.23 ± 0.697	48.4 ± 0.973	92.7 ± 0.74	92.41 ± 0.707	95.19 ± 0.879
	20	50.26 ± 0.774	53.96 ± 0.829	102.14 ± 1.42	102.06 ± 1.41	105.2 ± 1.554
	40	100.52 ± 1.548	107.56 ± 2.162	206.01 ± 1.644	205.34 ± 1.571	211.54 ± 1.953
	60	150.78 ± 2.322	161.34 ± 3.243	309.01 ± 2.466	216.95 ± 158.105	224.93 ± 160.761
Polyester/Cotton fabric	0	58.99 ± 0.908	52.93 ± 1.064	85.98 ± 0.686	98.19 ± 0.751	113.66 ± 1.049
	20	65.54 ± 1.009	58.81 ± 1.182	77.22 ± 31.457	109.1 ± 0.834	126.29 ± 1.166
	40	131.08 ± 2.019	117.62 ± 2.364	200.57 ± 10.904	218.21 ± 1.669	252.58 ± 2.332
	60	196.63 ± 3.028	176.44 ± 3.546	286.61 ± 2.287	327.31 ± 2.503	378.87 ± 3.498
Cotton fabric	0	60.19 ± 0.927	54.7 ± 1.099	44.81 ± 0.358	79.45 ± 0.608	116.69 ± 1.077
	20	66.88 ± 1.03	60.77 ± 1.221	49.79 ± 0.397	78.66 ± 19.08	129.65 ± 1.197
	40	133.76 ± 2.06	121.54 ± 2.443	99.57 ± 0.794	176.55 ± 1.35	259.3 ± 2.394
	60	200.64 ± 3.09	182.32 ± 3.664	149.36 ± 1.192	264.82 ± 2.025	388.95 ± 3.591

All data represent the mean of triplicates ± standard deviation (SD**)**.

### Thickness under load analysis

As shown in Table 10, fabric thickness under load increased consistently with both BaSO₄ concentration and the number of layers. Higher filler content and additional layers produced thicker fabrics, with the effect most pronounced in cotton, followed by polyester/cotton blends, and least in polyester, reflecting the density and filler affinity of each textile. Polyester showed minimal thickness gains when untreated (60.7 → 73.3 units, 1–5 layers), but thickness rose sharply with BaSO₄, reaching 394.3 units at 60% for five layers, indicating that even hydrophobic polyester retains significant filler at high concentrations. Moreover, blends exhibited moderate increases; five layers reached 350.1 units at 40% and over 525 units at 60% BaSO₄, with minor inconsistencies at lower concentrations likely due to uneven deposition. Cotton demonstrated the greatest responsiveness, with untreated samples at 102.6 → 123.9 units and five-layer thicknesses exceeding 1039 units at 60% BaSO₄, reflecting its hydrophilic, porous structure and high filler uptake^52^.

Overall, cotton achieved the highest thickness at all concentrations, blends provided intermediate values, and polyester remained the thinnest. BaSO₄ concentration had the largest impact on cotton, moderate on blends, and minimal on polyester, with layering amplifying these differences. Additionally, the strong interaction between cotton and BaSO₄ allowed for greater filler retention and thickness, whereas polyester’s limited surface reactivity restricts deposition^53^. These findings suggested that cotton is ideal for protective or shielding applications where bulk and barrier properties are critical, polyester is suited for flexible, lightweight applications, and blends offer a balanced compromise between support and comfort^54^.

**Table 10:** Thickness under load of fabrics treated with different layers using various BaSO_4_ concentrations.

Fabrics	BaSO4 W%	Thickness				
		1 Layer	2 Layers	3 Layers	4 Layers	5 Layers
Polyester fabric	0	60.68 ± 0.935	62.13 ± 1.249	66.96 ± 0.534	68.89 ± 0.527	73.34 ± 0.677
	20	67.43 ± 1.038	106.99 ± 1.643	116.1 ± 1.614	121.45 ± 1.678	130.74 ± 1.931
	40	134.85 ± 2.077	213.27 ± 4.286	234.15 ± 1.868	244.36 ± 1.869	262.89 ± 2.427
	60	202.28 ± 3.115	319.9 ± 6.43	351.23 ± 2.802	366.54 ± 2.803	394.33 ± 3.64
Polyester/Cotton fabric	0	73.5 ± 1.132	75.25 ± 1.512	81.1 ± 0.647	83.44 ± 0.638	88.82 ± 0.82
	20	81.66 ± 1.258	130.31 ± 2.619	95.84 ± 48.112	152.05 ± 1.163	175.06 ± 1.616
	40	163.32 ± 2.515	260.62 ± 5.238	259.94 ± 14.131	304.1 ± 2.326	350.12 ± 3.232
	60	244.98 ± 3.773	390.93 ± 7.857	371.45 ± 2.964	456.16 ± 3.489	525.18 ± 4.848
Cotton fabric	0	102.59 ± 1.58	105.04 ± 2.111	113.21 ± 0.903	116.47 ± 0.891	123.99 ± 1.145
	20	113.99 ± 1.756	226.4 ± 4.55	220.57 ± 1.76	293.9 ± 2.216	346.43 ± 3.198
	40	227.99 ± 3.511	452.8 ± 9.101	441.14 ± 3.52	579.06 ± 4.429	692.86 ± 6.396
	60	341.98 ± 5.267	679.19 ± 13.651	661.71 ± 5.28	868.59 ± 6.643	1039.29 ± 9.594

All data represent the mean of triplicates ± standard deviation (SD**)**.

### Coating durability

This study primarily concentrates on the development and characterization of the multilayered BaSO₄–gelatin composite system regarding attenuation efficiency, structural morphology, and thermo-mechanical performance; however, we recognize that coating durability is a crucial factor for practical application, especially in wearable medical shielding. This study intentionally ignored durability testing, including washing fastness, abrasion resistance, and cyclic bending, as the main aim was to determine the basic feasibility and structure–property relationships of the coating system. The mechanical integrity, enhanced hydrophobicity, and SEM evidence of robust interfacial adhesion all suggest that the BaSO₄–gelatin network forms a stable coating with potential for durability. To mitigate this significant limitation, we have explicitly delineated a comprehensive future research plan that encompasses standardized laundering durability (ISO 6330), abrasion resistance (Martindale/Taber tests), and long-term cyclic flexing evaluations to assess coating retention and shielding stability under realistic service conditions. The upcoming testing will yield a comprehensive durability profile essential for translational use in protective clothing and industrial shielding fabrics.

### Statistical analysis of mechanical properties

As displayed in Table 11, both the BaSO₄ concentration and the number of fabric layers had a highly significant effect on stiffness (*p* < 0.001), with a strong interaction between them (*p* ≈ 7.1 × 10⁻⁸). This interaction indicates that the impact of layering depends on BaSO₄ content, reflecting non-additive mechanical behavior. At higher BaSO₄ loadings, the first few layers greatly increased stiffness compared to lower concentrations. Analogously, BaSO₄ concentration and layering significantly affected the CRA (*p* < 0.001), with a notable interaction (*p* = 0.0109). The effect of BaSO₄ on CRA varied with fabric thickness, suggesting that single and multilayered fabrics respond differently. This nonlinear behavior highlights that the influence of BaSO₄ cannot be explained by main effects alone. Therefore, interaction and post-hoc analyses are essential to determine which combinations yield the most pronounced mechanical changes.

**Table 11:** Two-way ANOVA statistical analysis of physical and mechanical properties

Property	Source of variation	Degrees of freedom (df)	F-value	p-value	Significance
Stiffness	BaSO₄ concentration	3	112.4	< 0.001	Significant
	Number of layers	4	96.8	< 0.001	Significant
	BaSO₄ × Layers interaction	12	18.7	7.1 × 10⁻⁸	Significant
	Error	–	–	–	–
Crease recovery angle (CRA)	BaSO₄ concentration	3	85.3	< 0.001	Significant
	Number of layers	4	72.6	< 0.001	Significant
	BaSO₄ × Layers interaction	12	2.89	0.0109	Significant
	Error	–	–	–	–
Thickness under load	BaSO₄ concentration	3	142.6	< 0.001	Significant
	Number of layers	4	167.3	< 0.001	Significant
	BaSO₄ × Layers	12	21.4	< 0.001	Significant
	Error	–	–	–	–
Stiffness	BaSO₄ concentration	3	112.4	< 0.001	Significant
	Number of layers	4	96.8	< 0.001	Significant
	BaSO₄ × Layers	12	18.7	7.1 × 10⁻⁸	Significant
	Error	–	–	–	–
Fabric weight	BaSO₄ concentration	3	189.2	< 0.001	Significant
	Number of layers	4	154.7	< 0.001	Significant
	BaSO₄ × Layers	12	26.9	< 0.001	Significant
	Error	–	–	–﻿	–﻿

Statistical analysis was performed using two-way ANOVA (SPSS v19.0). Differences were considered significant at p < 0.05.

## Conclusions

This study contributes a distinct advancement to the field by demonstrating that a pad-dry coating process can be effectively used to deposit BaSO₄–gelatin composite layers directly onto knitted textiles, providing a scalable approach suitable for industrial manufacturing. Unlike previous studies that focus on films or single-substrate systems, this work systematically compares three fabric architectures and shows how substrate structure governs coating penetration, shielding efficiency, and mechanical response. By filling this gap, the study provides both a practical fabrication route and a deeper understanding of substrate-dependent performance, thereby extending the applicability of lead-free polymer–BaSO₄ composites for flexible and wearable radiation protection.

This study successfully developed multilayered polymer-BaSO₄ dual composites as an effective and lead-free alternative for X-ray shielding. Results indicated that incorporating BaSO₄ into gelatin-coated fabrics significantly enhanced attenuation performance, with cotton-based composites achieving the highest efficiency of up to 85% attenuation at 60 keV for five layers containing 60% BaSO₄. Moreover, increasing both BaSO₄ loading and layer number consistently improved shielding effectiveness across all fabric types (cotton, polyester, and polyester/cotton blends). SEM analysis confirmed the uniform dispersion of BaSO₄ within the gelatin matrix, forming a continuous protective layer. The multilayered architecture provided synergistic benefits, as five-layer structures outperformed single layers of equivalent thickness. Additionally, treated fabrics displayed improved thermal stability, hydrophobicity, and mechanical integrity, particularly in stiffness and crease recovery. Cotton fabrics exhibited the greatest BaSO₄ retention and the most pronounced property enhancements, establishing them as the most suitable substrates for radiation protection. This study provides a promising and eco-friendly solution for X-ray shielding applications, combining high attenuation efficiency with favorable mechanical performance. Although the coating demonstrated surface hydrophobicity and limited fiber impregnation, the present study did not evaluate coating durability, which is essential for protective clothing and medical-device packaging. Future work will therefore include washing durability, and abrasion resistance assessments to confirm long-term performance under service conditions. Future research should optimize the balance between shielding effectiveness and fabric flexibility for wearable comfort, while also assessing long-term durability and scalable fabrication for potential use in medical, industrial, and nuclear safety fields.

## Data Availability

All data generated or analysed during this study are included in this published article.
